# Beyond barriers: perceived barriers and winter sport participation intentions in a predominantly Zhejiang-based Chinese sample

**DOI:** 10.3389/fpsyg.2026.1902346

**Published:** 2026-10-19

**Authors:** QiZheng Zhou, ZhanDong Yang, DuoJie Zhang, YuJing Jiang

**Affiliations:** School of Sports Leisure and Tourism, Beijing Sport University, Beijing, China

**Keywords:** constraint negotiation, perceived barriers, self-efficacy, southern China, theory of planned behavior, winter sport participation

## Abstract

**Background:**

Following China's national strategy to promote winter sports, understanding the psychological mechanisms driving participation among residents in China's Yangtze River Delta region—a population with limited traditional exposure to ice and snow activities—has become increasingly important. This study integrated the Theory of Planned Behavior (TPB) with leisure constraint theory and self-efficacy theory to examine how perceived barriers were associated with winter sport participation intentions through attitudinal and behavioral control pathways, and whether self-efficacy moderates these relationships.

**Methods:**

A cross-sectional online survey was conducted with 415 self-identified residents of southern China. Structural equation modeling was employed to test the hypothesized relationships among perceived barriers, attitude, subjective norm, perceived behavioral control (PBC), self-efficacy, and participation intention. Bootstrap mediation analysis was used to assess indirect effects, and moderation analysis was performed to examine the conditional role of self-efficacy.

**Results:**

Perceived barriers demonstrated significant positive associations with both attitude (β = 0.16, *p* = 0.001) and PBC (β = 0.32, *p* < 0.001), a pattern consistent with constraint negotiation theory. Among the TPB predictors, PBC (β = 0.23, *p* < 0.001) and subjective norm (β = 0.20, *p* = 0.007) both significantly predicted participation intention, while attitude did not reach statistical significance (β = 0.01, *p* = 0.792). Self-efficacy exhibited the numerically largest standardized coefficient for participation intention (β = 0.323, *p* < 0.001). However, its coefficient did not differ significantly from those of PBC or subjective norm. The indirect association through PBC was significant (β = 0.076, 95% CI [0.028, 0.141]), whereas the indirect association through attitude was not significant (β = 0.002, 95% CI [– 0.014, 0.025]). The hypothesized moderating role of self-efficacy between perceived barriers and PBC was not supported.

**Conclusions:**

The positive associations of perceived barriers with attitude and PBC are inconsistent with a uniformly inhibitory interpretation of barriers, although the cross-sectional design and the absence of direct measures of constraint negotiation preclude conclusions about the underlying process. and underscore the importance of social influence and self-efficacy in shaping participation intentions. The independent predictive contribution of self-efficacy beyond PBC highlights the value of distinguishing these constructs in behavioral models. However, the cross-sectional design, convenience sampling, and geographic concentration of respondents limit causal inference and generalizability. These results offer theoretical and practical implications for promoting winter sport participation in non-traditional regions.

## 1 Introduction

The 2022 Beijing Winter Olympics catalyzed an unprecedented expansion of ice and snow sport participation across China. Between 2015 and 2022, the total market value of the national winter sport industry grew from approximately 270 billion to 800 billion Chinese yuan, reaching 890 billion yuan in 2023. During the 2023 to 2024 season, winter leisure tourism generated an estimated 524.7 billion yuan in revenue, with participation reaching 430 million person-visits (Cui et al., 2026). Nationwide, the number of ski resorts reached 646 by 2017, with indoor ski resorts expanding rapidly across southern provinces as part of the national “southward expansion, westward advance” strategy (Wang et al., 2019). These figures signal a structural transformation in the Chinese sport consumption landscape, positioning winter sports as a rapidly expanding segment of the national leisure economy.

A particularly striking dimension of this growth has been its geographic diffusion into southern China. Under the national policy of expanding winter sports to the south, west, and east, every province and municipality south of the Qinling-Huaihe line had established at least one indoor or outdoor ski facility by 2024, totaling 131 venues. Participation data corroborate this infrastructure expansion: southern skiers accounted for 34.39% of total national ski visits during the 2023 to 2024 season, with year-on-year increases of 9.32% in the South China region and 6.10% in the Southwest. These trends indicate that southern residents have emerged as a consequential demographic segment in the Chinese skiing market, fundamentally reshaping the geographic contours of winter sport participation.

Despite this remarkable growth, winter sport participation among southern Chinese residents faces distinctive challenges. Southern residents typically lack the natural snow conditions, cultural traditions, and socialization opportunities that facilitate winter sport engagement in northern regions. They face structural barriers including geographic distance from natural ski venues, elevated economic costs, and limited access to specialized instructional resources. These barriers are particularly acute given that winter sports require significant skill investment and equipment access relative to many other leisure activities. Investigations conducted in Guangzhou, Shenzhen, and other southern cities have found that structural constraints, specifically economic costs and geographic distance, exert the strongest inhibitory effects on southern residents' winter sport participation intentions (Chen et al., 2026; Deng and Zhou, 2022). Consistent with Ntovoli et al. (2024), structural constraints represent the most prominent barriers for sport participants.

Beyond the material and infrastructural barriers, winter sports offer distinctive experiential qualities that warrant consideration. Winter sports are typically experienced in natural or semi-natural environments with distinctive landscape characteristics—snow-covered mountains, alpine settings, and open-air venues—that may carry psychological and restorative value beyond that of conventional indoor exercise. Emerging evidence suggests that perceived exposure to natural environments, including green and blue spaces, is associated with mental health benefits through cultural ecosystem services (Guo et al., 2026), and that nature-based recreation can contribute to psychological well-being through multiple mechanistic pathways (Bratman et al., 2019). The intersection of physical activity, natural environment exposure, and psychological restoration represents a potentially important dimension of winter sport participation that has received limited attention in the existing literature on sport participation intentions.

Despite the practical significance of winter sport expansion into southern China, the existing body of research on winter sport participation behavior has relied predominantly on samples drawn from traditional winter sport regions in northern and northeastern China. These studies have focused on skier satisfaction and participation willingness among populations that possess prior exposure to winter sport culture. The specific psychological mechanisms through which southern residents—who must actively seek out winter sport opportunities rather than encountering them as part of their natural environment—form participation intentions have received limited empirical attention, with the available evidence largely confined to qualitative or descriptive analyses rather than quantitative hypothesis testing.

Furthermore, the canonical Theory of Planned Behavior (TPB; Ajzen, 1991) does not incorporate perceived barriers as an exogenous antecedent variable, thereby failing to capture the mechanisms through which barriers may relate to attitude and behavioral control in high-barrier contexts (Gao et al., 2026). The TPB also does not account for the potential role of self-efficacy as a distinct cognitive resource beyond perceived behavioral control, particularly in contexts requiring substantial skill acquisition.

To address these gaps, the present study targeted urban residents in southern China and adopted an extended TPB framework that integrates leisure constraint theory and self-efficacy theory. Specifically, the study sought to elucidate how perceived barriers pertaining to economic costs, geographic distance, and skill deficiencies relate to winter sport participation intention through the mediating pathways of attitude and PBC, and to test whether self-efficacy moderates the barrier-to-PBC link. In doing so, the study aimed to advance understanding of the cognitive mechanisms governing winter sport participation decisions in non-traditional regions and to inform evidence-based strategies for promoting winter sport engagement among underserved populations.

## 2 Theoretical framework and hypothesis development

### 2.1 Leisure constraint theory, constraint negotiation, and perceived barriers

Complementing the motivational emphasis of the TPB, leisure constraint theory provides a conceptual apparatus for understanding barriers to participation. Crawford and Godbey (1987) proposed that factors that intervene in or inhibit leisure engagement can be categorized into three classes: intrapersonal constraints (individual psychological states that affect leisure preferences), interpersonal constraints (social factors that affect the ability to find compatible partners for leisure activities), and structural constraints (external factors that intervene between preference and participation, such as cost, time, and accessibility). This foundational taxonomy was subsequently extended through the development of a hierarchical model in which intrapersonal constraints are encountered first, followed by interpersonal constraints, and finally structural constraints (Crawford et al., 1991). Empirical work at ski resorts in northeastern China has confirmed the applicability of the constraint framework to skiing behavior (Liu, 2024), while research in the sport tourism domain has highlighted spatial barriers such as travel distance and physical barriers such as fitness requirements as particularly salient constraints (Hinch et al., 2005).

A critical theoretical development within the leisure constraints literature is the emergence of constraint negotiation theory. Jackson et al. (1993) argued that leisure constraints do not necessarily suppress participation; rather, individuals may deploy negotiation strategies to overcome obstacles. Participation therefore depends not on the absence of constraints but on successful negotiation through them, which can modify rather than foreclose engagement. This represents a fundamental conceptual shift from the earlier framework, which had emphasized the inhibitory function of constraints, toward a more dynamic perspective in which constraints and negotiation efforts coexist and interact. Empirical investigations have demonstrated that efforts to overcome leisure constraints significantly increase leisure participation, and that the negotiation process consistently mediates the constraint-participation relationship (Kim et al., 2022). It is important to note that constraint negotiation theory does not claim that all individuals successfully negotiate constraints, nor that constraint perception invariably leads to negotiation effort. Rather, the theory emphasizes that the relationship between constraints and participation is mediated by the degree to which individuals engage in negotiation strategies—a construct distinct from constraint perception itself. This distinction is central to the present study: while perceived barriers were measured, specific negotiation strategies, coping mechanisms, or resource mobilization behaviors were not directly assessed.

Subsequent empirical work has demonstrated that among motivated individuals, heightened barrier awareness can be accompanied by stronger participation-related cognitions. In the context of residents of southern China, who must actively seek out winter sport opportunities, the process of identifying and appraising barriers may be associated with coping mechanisms that relate to both evaluative orientation and perceived controllability. This interpretation is consistent with the hierarchical leisure constraints framework, which posits that intrapersonal and interpersonal constraints carry greater explanatory weight than structural constraints in shaping leisure participation decisions (Pizzo et al., 2023). However, the cross-sectional design of the present study precludes direct assessment of the temporal ordering of barrier perception and cognitive change. Building on this constraint negotiation rationale, the following hypotheses were proposed.

H1a: Perceived barriers are positively associated with attitude toward winter sport participation among residents of the study region.

H1b: Perceived barriers are positively associated with perceived behavioral control regarding winter sport participation among residents of the study region.

### 2.2 The theory of planned behavior and winter sport participation

Understanding the psychological determinants of sport participation intentions has long been a central concern in sport and exercise psychology. The TPB (Ajzen, 1991) represents perhaps the most widely applied framework for predicting exercise-related intentions and behavior. According to the TPB, behavioral intention is jointly determined by three cognitive antecedents: attitude toward the behavior, subjective norm, and PBC. A landmark meta-analysis synthesizing 72 studies involving over 21,000 participants confirmed the robust predictive validity of these three variables for exercise intentions, with PBC exhibiting the largest mean effect size (β ≈ 0.46; Hagger et al., 2002). More recent meta-analytic evidence has continued to support these predictive relationships, with attitudes, subjective norms, and PBC demonstrating significant direct effects on intention in health behavior contexts (Hagger et al., 2022). Subsequent work has demonstrated that the TPB can be meaningfully extended to domain-specific contexts; for instance, incorporating environmental awareness into the TPB framework significantly enhanced its explanatory power for ski destination choice among Italian skiers Bae et al., (2020).

Within the specific context of winter sport participation, the applicability of the TPB has received targeted empirical scrutiny. (Bae et al. 2020) validated an extended TPB model for adolescent participation in non-traditional sports, confirming that attitude, subjective norm, and PBC positively predict participation intention, with prior knowledge enhancing attitude and intention. In non-traditional winter sport regions, intention formation appears to depend substantially on attitude activation and social norm transmission. When individuals lack direct experiential contact with ice and snow activities, media exposure, school-based curricula, and peer modeling can reduce perceived psychological distance and cultural unfamiliarity.

A substantial body of empirical evidence has validated the predictive utility of the TPB across diverse sport and exercise settings, while also revealing systematic variation in the relative magnitude of each predictor. Meta-analytic evidence from predominantly Western samples indicates that attitude typically exhibits the strongest association with exercise intention (mean β ≈ 0.42 to 0.51), whereas subjective norm, though consistently significant, tends to show the weakest effect (β ≈ 0.18 to 0.22; Hagger et al., 2002). However, the relative weighting of TPB predictors appears to vary across cultural contexts. Empirical evidence from Chinese university student samples demonstrates that subjective norm consistently exerts a significant positive effect on exercise intention, with standardized path coefficients ranging from 0.238 to 0.239 across different model specifications (Feng et al., 2022). Moreover, the predictive strength of subjective norm is contingent upon cultural value orientation: among individuals with high levels of horizontal or vertical collectivism, the effect of subjective norm on intention is markedly stronger (β = 0.316 to 0.349) than among those with low collectivism (β = 0.221 to 0.233; Feng et al., 2023). These findings suggest that the default hierarchy of TPB predictors established in predominantly Western research programs may not generalize to East Asian cultural contexts.

Applying the TPB to winter sport participation among southern residents requires careful consideration of the unique psychological dynamics at play. For a population with minimal prior exposure to ice and snow environments, winter sport participation constitutes a prototypically novel, high-barrier behavior, in which the formation of intention depends on the interplay among cognitive evaluations. Attitude captures the affective and evaluative orientation toward participation, encompassing anticipated health benefits, recreational value, and perceptions of challenge. Subjective norm reflects the perceived expectations and approval of significant referents, including family members, peers, coaches, and social media contacts. PBC encompasses the individual's sense of mastery over the requisite skills, access to resources such as facilities, equipment, and time, and confidence in overcoming situational obstacles. For southern residents, who characteristically lack the natural snow conditions and cultural immersion that facilitate winter sport socialization, positive attitudes may serve to activate intrinsic motivation, social norms may furnish behavioral legitimacy and vicarious modeling, and high PBC may attenuate the environmental unfamiliarity and skill-related anxiety associated with novel sport contexts. On the basis of the foregoing theoretical and empirical considerations, the following hypotheses were advanced:

H2a: Attitude is positively associated with winter sport participation intention.

H2b: Subjective norm is positively associated with winter sport participation intention.

H2c: Perceived behavioral control is positively associated with winter sport participation intention.

### 2.3 Self-efficacy and its distinction from perceived behavioral control

Although PBC and self-efficacy are frequently discussed in tandem within the sport participation literature, these constructs are theoretically and operationally distinct. PBC, rooted in the TPB, reflects the individual's overall appraisal of the ease or difficulty of performing a target behavior, encompassing both external resource availability and internal capability assessments (Ajzen, 1991). Self-efficacy, grounded in social cognitive theory, refers specifically to the individual's confidence in their capacity to organize and execute the behavioral sequences required for successful performance (Bandura, 1986). Ajzen (2002) subsequently argued from an integrative perspective that PBC comprises two separable dimensions: controllability, which pertains to external conditions, and self-efficacy, which pertains to internal capability beliefs.

Empirical evidence has confirmed that self-efficacy and PBC are empirically separable constructs, with each contributing unique variance to the prediction of intention and behavior in health contexts (Parkinson et al., 2017). Further studies have demonstrated that self-efficacy is more predictive of behavioral intentions and actual behavior than perceived control, supporting the need to treat them as distinct rather than interchangeable constructs (Povey et al., 2000). When these dimensions are explicitly differentiated in measurement and modeling, self-efficacy may function not merely as a subcomponent of PBC but as a higher-order cognitive resource.

Empirical work in sport and exercise psychology has examined whether self-efficacy may function as a boundary condition for the effects of perceived barriers on control-related cognitions. Early evidence suggested that individuals with higher self-efficacy were better able to maintain robust behavioral control perceptions in the face of perceived obstacles (Terry and O'Leary, 1995). Subsequent analyses among university student samples indicated that self-efficacy positively moderated the relationship between external constraints and control perceptions (Rhodes and Courneya, 2003). Cross-cultural investigations have further suggested that self-efficacy may strengthen the conversion of barrier appraisals into active control cognitions across diverse cultural backgrounds and sport types (Hagger et al., 2010). On the basis of the foregoing evidence, the following hypothesis was proposed:

H2d: Self-efficacy is positively associated with winter sport participation intention.

H3: Self-efficacy moderates the relationship between perceived barriers and PBC, such that the positive association of perceived barriers with PBC is stronger at higher levels of self-efficacy.

Additionally, given that self-efficacy was included as a direct predictor of participation intention in the structural model, its direct path to intention requires theoretical justification. Self-efficacy is theorized to influence intention through multiple mechanisms: by enhancing outcome expectations, by reducing perceived difficulty of the target behavior, and by increasing persistence in the face of obstacles (Bandura, 1986). In the context of winter sport participation among populations without traditional exposure to these activities, self-efficacy beliefs may be particularly consequential because individuals must draw on internal capability assessments in the absence of direct experiential knowledge. The structural model is presented in Figure 1.

**Figure 1:**
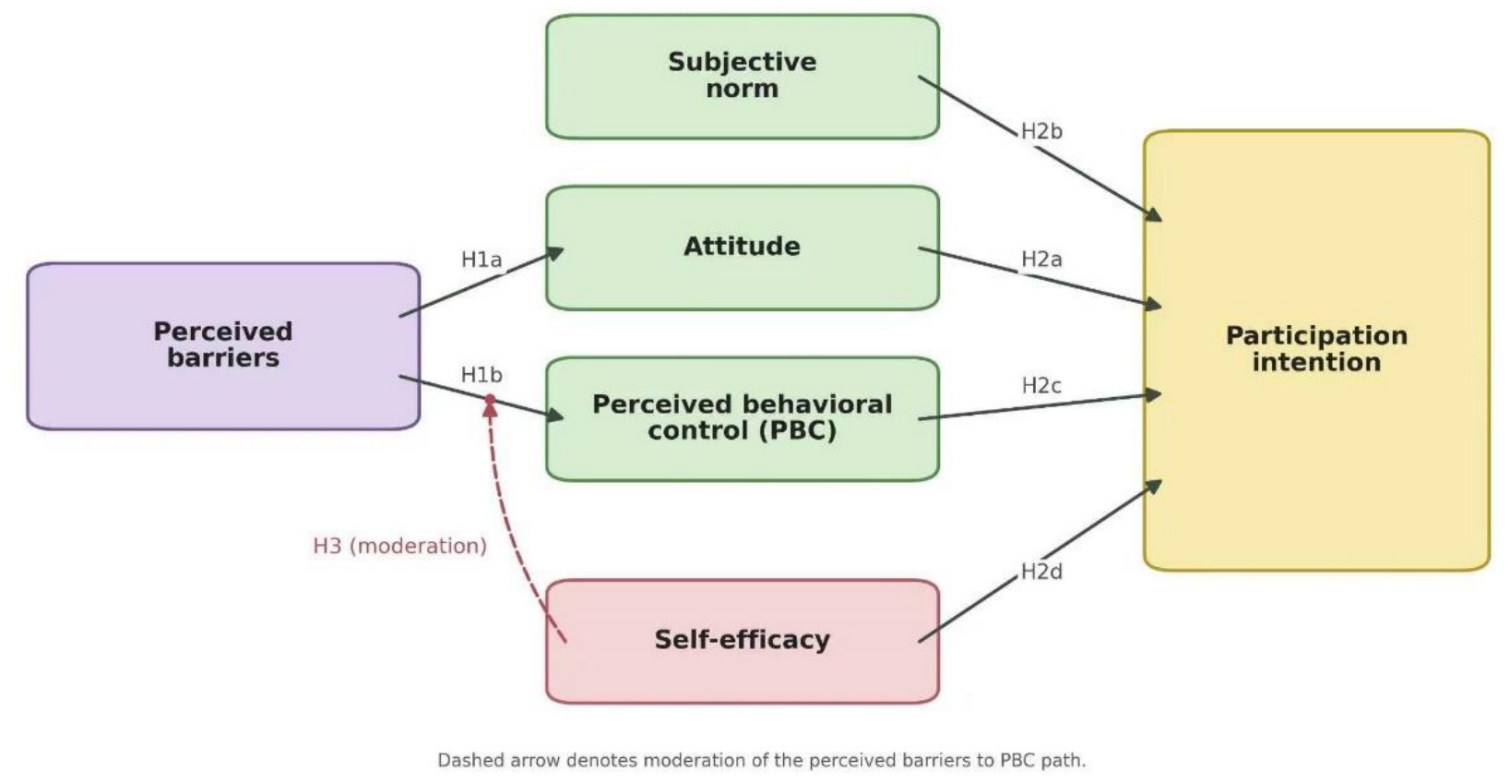
Conceptual model.

## 3 Method

### 3.1 Participants and procedure

Participants were residents of southern China, defined as individuals whose primary residence was located south of the Qinling Mountains and Huaihe River geographic boundary. Data were collected between March 15 and March 18, 2026 via the Wenjuanxing online survey platform (www.wjx.cn). A convenience sampling strategy was employed, with participant recruitment conducted through the social media platforms Douyin and WeChat. Respondents were encouraged to forward the survey link to their personal social networks to maximize reach (snowball sampling). The first questionnaire item asked respondents whether they were residents of southern China. Respondents who selected “no” were automatically directed to the end of the survey and did not complete the substantive items. Participants who completed the questionnaire were eligible to receive a monetary incentive of up to five yuan.

The study protocol was approved by the Institutional Review Board of Beijing Sport University before data collection (**2026233H**). All participants provided informed electronic consent prior to commencing the survey. All procedures were conducted in accordance with the Declaration of Helsinki. Participants were informed of the voluntary nature of participation, their right to withdraw at any point without penalty, and the measures in place to ensure data confidentiality. Survey responses were collected anonymously, with no personally identifying information (e.g., name, ID number, and IP address) recorded. Data were stored on password-protected servers accessible only to the research team.

#### 3.1.1 Sample size justification

Although a formal a priori power analysis was not conducted—a limitation discussed in Section 5.6—the final analytic sample of 415 cases exceeds commonly recommended minimum sample sizes for structural equation modeling. No formal a priori power analysis was conducted. A sensitivity analysis indicated that, with *N* = 415, α = 0.05, and 80% power, the moderation analysis could detect an incremental effect of approximately *f* ^2^ = 0.019 or larger. The study may therefore have had limited sensitivity to interaction effects smaller than this value. Given that the present structural model involved six latent constructs with 22 observed indicators, the achieved sample size was deemed sufficient for obtaining stable parameter estimates, though readers should interpret the results with appropriate caution regarding statistical power for detecting smaller effects, particularly for the interaction term in the moderation analysis.

A total of 522 responses were initially collected. Of these, 17 cases from respondents residing outside southern provinces were removed based on the screening question. An additional 90 cases were excluded through reliability screening procedures that involved: A total of 522 responses were initially recorded. Seventeen respondents who indicated that they were not residents of southern China were excluded by the screening item. Among the remaining 505 respondents, 90 failed the embedded attention check by not selecting the instructed response option. The final analytic sample therefore comprised 415 respondents, corresponding to 79.5% of the initial records.

#### 3.1.2 Demographic and geographic characteristics

A total of 415 valid responses were collected. Demographic characteristics are summarized in Table 1. The sample was gender-balanced (46.51% male, 53.49% female). TParticipants aged 19–25 accounted for the largest proportion (33.73%, 140 participants), serving as the primary sample group. Respondents aged 26–30, 31–35 and 36–40 constituted the middle sample proportion, accounting for 16.14%, 19.04% and 13.98% respectively. By contrast, the groups aged 18 and below (2.41%), 41–50 (9.40%) and over 50 (5.30%) occupied relatively small proportions. Overall, the age distribution covered diverse groups with a rational structure, ensuring sample representativeness and providing valid data support for subsequent empirical analysis.

**Table 1:** Means, standard deviations, cronbach's alpha, and intercorrelations among study variables (*N* = 415).

Variables	*M*	SD	α	1	2	3	4	5	6
1. PB	5.24	1.45	0.851	—					
2. ATT	5.61	1.40	0.925	0.160^**^	—				
3. SN	5.29	1.42	0.804	0.246^**^	0.661^**^	—			
4. PBC	5.14	1.47	0.858	0.324^**^	0.376^**^	0.733^**^	—		
5. SE	5.42	1.48	0.916	0.210^**^	.387^**^	0.509^**^	0.413^**^	—	
6. INT	5.11	1.67	0.944	0.181^**^	0.357^**^	0.541^**^	0.516^**^	0.525^**^	—

*p* < 0.01 (two-tailed).

All scales are 7-point Likert scales (1 = strongly disagree, 7 = strongly agree).

PB, Perceived Barriers; ATT, Attitude; SN, Subjective Norm; PBC, Perceived Behavioral Control; SE, Self-Efficacy; INT, Participation Intention.

^*^Statistically significant; ^**^Highly statistically significant.

This youthful profile reflects the physical demands of skiing and the broader appeal of snow sports among younger demographics. Education levels were high: bachelor's degree holders constituted the largest group (44.03%), followed by associate degrees (20.90%) and postgraduate qualifications (14.18%); those with high school or below accounted for 20.97%. By occupation, students (32.34%) and corporate employees (27.36%) predominated, with professionals and technicians comprising 15.43%—collectively representing over 70% of the sample. The largest monthly income bracket was < ¥2,000 (39.80%), consistent with the high proportion of students; the >¥8,000 bracket followed (20.90%), with the remaining income categories distributed relatively evenly. In terms of skiing frequency, the majority of participants (70%) reported skiing 3–5 times in the preceding 12 months, with 6–10 times accounting for 15%, 11–20 times for 10%, and high-frequency skiers (>20 times) for 5%. This distribution is consistent with the recreational pattern typical of southern China's skiers, whose access is largely confined to weekend visits at indoor snow facilities.

IP-derived access locations indicated that 279 responses (67.23%) were submitted from Zhejiang Province. These locations reflect where the questionnaire was accessed and should not be interpreted as verified permanent residence. Given this geographic concentration, the study population may be more accurately characterized as residents of the Yangtze River Delta region (encompassing Zhejiang, Shanghai, Jiangsu, and surrounding areas), rather than as representative of southern China as a whole. This limitation is discussed further in Section 5.6.

Given that skiing is a relatively high-investment sport with particular appeal among higher-income populations in the Yangtze River Delta region, and given the rapid recent growth in indoor and outdoor ski facilities in this area, the preponderance of Zhejiang-based respondents was considered to reflect both demand-side preferences and supply-side infrastructure developments. Nevertheless, the geographic concentration limits the generalizability of findings to other southern provinces with different economic profiles, infrastructure availability, and cultural orientations toward winter sports.

### 3.2 Measures

The survey instrument was developed by adapting established scales to the winter sport participation context. Six core constructs were measured. Perceived barriers were assessed using four items adapted from the leisure constraint scale developed by Alexandris and Carroll (1997). These items addressed structural constraints including economic costs, geographic distance, time requirements, and skill demands. Attitude, subjective norm, and perceived behavioral control were operationalized in accordance with the guidelines outlined in the TPB measurement manual (Ajzen, 1991), employing seven-point Likert-type semantic differential scales. Attitude was measured using four items, subjective norm using three items, and PBC using four items. Self-efficacy items were developed based on the guidelines for constructing context-specific self-efficacy scales (Bandura, 2006). Three items assessed participants' confidence in their ability to participate in winter sports under varying conditions. Participation intention items were constructed in adherence to the Target, Action, Context, and Time (TACT) principle recommended by Ajzen (1991). Four items assessed participants' intention to participate in winter sports within the upcoming winter season. The final instrument comprised 22 items measured on seven-point Likert scales (1 = strongly disagree, 7 = strongly agree).

#### 3.2.1 Scale adaptation and translation procedures

All scales were originally developed in English. The adaptation process involved the following steps: First, the first author translated all items from English to Chinese. Second, a bilingual expert in sport psychology who was not involved in the study back-translated the Chinese items into English. Third, the original and back-translated versions were compared, and discrepancies were resolved through discussion between the translators. Fourth, the Chinese version was reviewed by two experts in sport psychology for content validity and cultural appropriateness. Fifth, a pilot test was conducted with a convenience sample of 30 residents of southern China to assess item clarity, response variability, and completion time. Minor wording adjustments were made following the pilot test to improve comprehensibility. No items were added or removed as a result of this process.

### 3.3 Common method bias

Because all variables were assessed through self-report in a single survey, common method variance was considered a potential concern. Harman's unrotated factor analysis indicated that the first factor accounted for 40.24% of the total variance. In addition, a single-factor CFA model demonstrated poor fit, χ^2^(209) = 3815.71, *p* < 0.001, CFI = 0.487, TLI = 0.433, RMSEA = 0.204, 90% CI [0.199, 0.210], and SRMR =0.154. These results indicate that the covariance among the measures was not adequately represented by a single common factor. Nevertheless, these diagnostic tests cannot fully exclude common method variance.

At the statistical level, two approaches were employed to assess the potential influence of common method variance. First, Harman's single-factor test was conducted by submitting all 22 scale items to an unrotated principal component analysis. The first component accounted for 40.27% of the total variance, which is below the conventional 50% threshold, providing preliminary evidence that common method bias may not pose a severe threat to the validity of the findings. However, it should be noted that Harman's single-factor test has been extensively criticized for its limited sensitivity and inability to adequately detect common method variance (Podsakoff et al., 2003). As a supplementary check, a common latent factor approach was employed: a CFA model was specified in which all items loaded onto both their intended constructs and an unmeasured common latent factor.

## 4 Results

### 4.1 Descriptive statistics and correlations

Descriptive statistics and bivariate correlations for all study variables are presented in Table 1. Construct means ranged from 5.11 to 5.61 on the seven-point scale, and standard deviations ranged from 1.40 to 1.67, indicating adequate response variability. All Cronbach's alpha coefficients exceeded the 0.70 benchmark, ranging from 0.804 to 0.944, providing evidence of satisfactory internal consistency. The correlation matrix revealed that all pairwise associations were positive and statistically significant (ps < 0.01). The highest correlation was observed between subjective norm and PBC (*r* = 0.733), while the remaining coefficients ranged from 0.160 to 0.661.

### 4.2 Confirmatory factor analysis

A six-factor measurement model was evaluated using maximum likelihood estimation. The model demonstrated acceptable fit to the data, χ^2^(194) = 615.81, *p* < 0.001, CFI = 0.940, TLI = 0.929, RMSEA = 0.072, 90% CI [0.066, 0.079], and SRMR = 0.064, GFI = 0.915. All fit indices met or approached conventional thresholds (CFI/TLI > 0.90; RMSEA < 0.08), indicating satisfactory overall model-data correspondence.

With respect to convergent validity, all standardized factor loadings ranged from 0.592 to 0.904 and were statistically significant at the *p* < 0.001 level (Table 2). Average variance extracted (AVE) values ranged from 0.591 to 0.809, exceeding the 0.50 criterion, and composite reliability (CR) values ranged from 0.812 to 0.944, surpassing the 0.70 threshold. These results collectively confirmed adequate convergent validity.

**Table 2:** Confirmatory factor analysis: factor loadings, average variance extracted, and composite reliability (*N* = 415).

Construct	Item	Std. loading (λ)	AVE	CR	α
PB	PB1	0.676	0.592	0.851	0.851
	PB2	0.680			
	PB3	0.850			
	PB4	0.852			
ATT	ATT1	0.826	0.757	0.926	0.925
	ATT2	0.867			
	ATT3	0.882			
	ATT4	0.904			
SN	SN1	0.709	0.592	0.812	0.804
	SN2	0.816			
	SN3	0.779			
PBC	PBC1	0.581	0.616	0.863	0.858
	PBC2	0.881			
	PBC3	0.832			
	PBC4	0.824			
SE	SE1	0.860	0.786	0.917	0.916
	SE2	0.895			
	SE3	0.905			
INT	INT1	0.885	0.809	0.944	0.944
	INT2	0.913			
	INT3	0.902			
	INT4	0.897			

All factor loadings are significant at *p* < 0.001.

AVE, Average Variance Extracted (criterion: > 0.50); CR, Composite Reliability (criterion: > 0.70).

#### 4.2.1 Discriminant validity

Discriminant validity was assessed using two complementary approaches. First, the Fornell-Larcker criterion was evaluated (Table 3). The square root of the AVE for each construct exceeded all corresponding inter-construct correlations. Notably, the highest inter-construct correlation was between subjective norm and PBC (*r* = 0.733), yet the square root of AVE for subjective norm (√AVE = 0.769) remained larger than this value, satisfying the Fornell-Larcker requirement.

**Table 3:** Fornell-Larcker criterion matrix for discriminant validity (*N* = 415).

	1. PB	2. ATT	3. SN	4. PBC	5. SE	6. INT
1. PB	**0.769**					
2. ATT	0.160	**0.870**				
3. SN	0.246	0.661	**0.769**			
4. PBC	0.324	0.376	0.733	**0.785**		
5. SE	0.210	0.387	0.509	0.413	**0.887**	
6. INT	0.181	0.357	0.541	0.516	0.525	**0.899**

Bold diagonal values represent the square root of AVE (√AVE) for each construct.

Second, given concerns regarding the sensitivity of the Fornell-Larcker criterion to detect discriminant validity problems (Henseler et al., 2015), heterotrait-monotrait (HTMT) ratios of correlations were calculated for each pair of constructs. HTMT values ranged from 0.181 to 0.884. The HTMT ratio between self-efficacy and PBC was 0.466, providing clear evidence that the two constructs were empirically distinguishable. The highest value was observed between subjective norm and PBC, HTMT = 0.884. Although this value exceeded the conservative 0.85 criterion, it remained below the 0.90 threshold commonly applied to conceptually related constructs. Detailed data are presented in Table 4. The six-factor model was compared with an alternative five-factor model in which the self-efficacy and PBC items loaded on a single factor. The alternative model demonstrated substantially poorer fit, χ^2^(199) = 1326.58, CFI = 0.840, TLI = 0.814, RMSEA = 0.117, 90% CI [0.111, 0.123], and SRMR = 0.093. The deterioration in fit was substantial, Δχ^2^(5) = 710.77, *p* < 0.001, ΔCFI = 0.100, supporting the empirical distinction between self-efficacy and PBC.

**Table 4:** Heterotrait-monotrait (HTMT) ratios (*N* = 415).

	1. PB	2. ATT	3. SN	4. PBC	5. SE	6. INT
1. PB	—					
2. ATT	0.181	—				
3. SN	0.299	0.764	—			
4. PBC	0.877	0.423	0.884	—		
5. SE	0.238	0.421	0.593	0.466	—	
6. INT	0.202	0.382	0.620	0.574	0.564	—

HTMT, Heterotrait-Monotrait ratio of correlations.

Discriminant validity is established if all HTMT values are lower than 0.85.

### 4.3 Structural model and hypothesis testing

The structural model results are depicted in Figure 2, and the full set of path coefficients is summarized in Table 5. Consistent with the constraint negotiation rationale, perceived barriers exhibited significant positive associations with both attitude (β = 0.16, *p* = 0.001) and PBC (β = 0.32, *p* < 0.001), supporting H1a and H1b. Regarding the predictive effects of the TPB core variables on participation intention, PBC (β = 0.23, *p* < 0.001) and subjective norm (β = 0.20, *p* = 0.007) both demonstrated significant positive associations with intention, supporting H2c and H2b, respectively. Among the three canonical TPB variables, PBC exhibited the numerically largest standardized coefficient for participation intention, followed closely by subjective norm. The coefficients for PBC and subjective norm did not differ significantly, *p* = 0.745. However, a formal test comparing the magnitudes of these two coefficients was not conducted, and the possibility that they do not differ statistically cannot be ruled out. Attitude did not significantly predict intention (β = 0.01, *p* = 0.792), and H2a was therefore not supported. Self-efficacy exhibited the numerically largest standardized coefficient for participation intention. However, formal coefficient comparisons indicated that its coefficient did not differ significantly from that of PBC, *p* = 0.242, or subjective norm, *p* = 0.172. The baseline TPB model (attitude, subjective norm, and PBC) accounted for 32.5% of the variance in participation intention (*R*^2^ = 0.325). The inclusion of self-efficacy increased the explained variance to 40.2% (Δ*R*^2^ = 0.076), confirming the incremental predictive contribution of self-efficacy beyond the canonical TPB variables.

**Figure 2:**
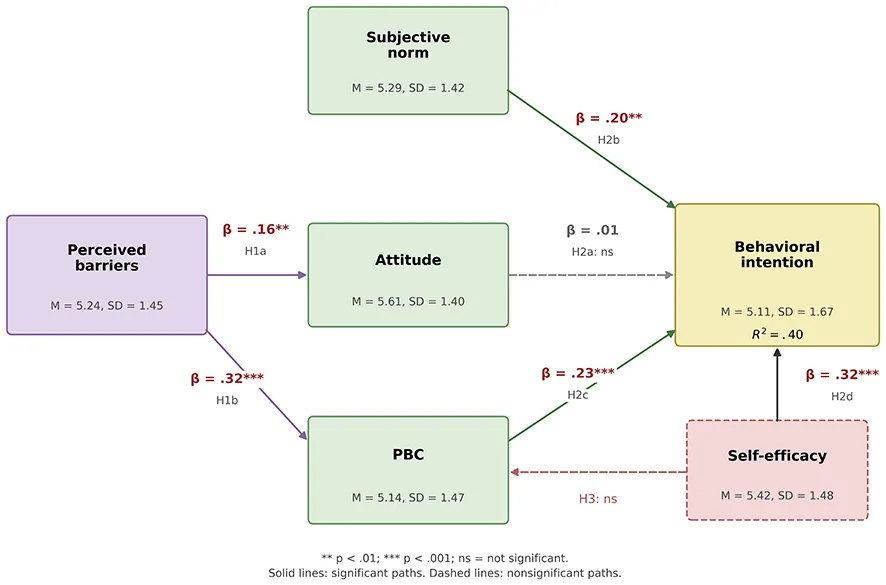
Structural model results with standardized path coefficients (*N* = 415). Solid lines indicate significant paths; dashed lines indicate nonsignificant paths. *p* < 0.01; *p* < 0.001; ns, not significant. ✓, hypothesis supported; *X*, hypothesis not supported.

**Table 5:** Structural model path coefficients and hypothesis testing results (*N* = 415).

Hypothesis	Path	β	SE	*t*	*p*	95% CI LL	95% CI UL
H1a	PB → ATT	0.160^**^	0.049	30.30	0.001	0.065	0.256
H1b	PB → PBC	0.324^***^	0.047	6.95	< 0.001	0.232	0.415
H2a	ATT → INT	0.014	0.052	0.26	0.792	−0.089	0.116
H2b	SN → INT	0.196^**^	0.073	2.69	0.007	0.053	0.338
H2c	PBC → INT	0.234^***^	0.058	4.07	< 0.001	0.121	0.347
H2d	SE → INT	0.323^***^	0.045	7.24	< 0.001	0.236	0.411

β, standardized coefficient; SE, standard error; CI, confidence interval.

^**^p < 0.01; ^***^p < 0.001.

### 4.4 Mediation analysis

The indirect effects of perceived barriers on participation intention through attitude and PBC were examined using bootstrap resampling with 5,000 iterations and bias-corrected 95% confidence intervals (CIs; Table 6). T The indirect association through attitude was not significant, β = 0.002, Boot SE = 0.009, 95% CI [−0.014, 0.025]. The indirect association through PBC was significant, β = 0.076, Boot SE = 0.029, 95% CI [0.028, 0.141]. The total indirect association was 0.078, Boot SE =0.032, 95% CI [0.024, 0.150]. These results indicate that perceived barriers were indirectly associated with participation intention through the behavioral control pathway but not through the attitudinal pathway. It is important to note that the cross-sectional design of this study precludes causal interpretation of these indirect effects; the reported mediation pathways reflect statistical associations consistent with the hypothesized model structure but do not establish temporal precedence or causal mechanisms.

**Table 6:** Bootstrap mediation analysis results (bootstrap resamples = 5,000; *N* = 415).

Mediation path	a path β	b path β	Indirect Effect	Boot SE	95% CI LL	95% CI UL	Significance
PB → ATT → INT	0.160	0.014	0.002	0.009	−0.014	0.025	Not significant
PB → PBC → INT	0.324	0.234	0.076^*^	0.029	0.028	0.141	Significant
**Total parallel indirect**	**—**	**—**	**0.078** ^*^	**0.032**	**0.024**	**0.150**	**Significant**

Bootstrap resamples, 5,000; 95% CI, bias-corrected confidence interval.

^*^95% CI excluding zero.

LL, Lower Limit; UL, Upper Limit.

The cross-sectional design precludes causal interpretation of mediation pathways; results reflect statistical associations consistent with the hypothesized model structure.

### 4.5 Moderation analysis

Hierarchical regression was employed to test whether self-efficacy moderated the relationship between perceived barriers and PBC (Table 7). All continuous variables were mean-centered prior to the computation of interaction terms to reduce multicollinearity. In Model 1, perceived barriers alone significantly predicted PBC (β = 0.324, *p* < 0.001, *R*^2^ = 0.105). In Model 2, the addition of self-efficacy significantly improved model fit (Δ*R*^2^ = 0.124, *p* < 0.001), and self-efficacy exhibited a strong positive main effect on PBC (β = 0.361, *p* < 0.001). In Model 3, the interaction term between perceived barriers and self-efficacy was entered; its coefficient did not reach statistical significance (β = −0.064, *p* = 0.151), and model explanatory power did not increase appreciably (Δ*R*^2^ = 0.004, *p* = 0.151). Accordingly, H3 was not supported.

**Table 7:** Hierarchical regression analysis for moderating effect of self-efficacy on PB-PBC relationship (DV: PBC; *N* = 415).

Variable	Model 1 β	Model 2 β	Model 3 β
Perceived barriers (PB)	0.324^***^	0.248^***^	0.237^***^
Self-efficacy (SE)	—	0.361^***^	0.350^***^
PB × SE (Interaction)	—	—	–.064
*95% CI LL*	0.215	0.150	0.142
*95% CI UL*	0.433	0.351	0.340
*R^2^*	0.105	0.229	0.233
*ΔR^2^*	—	0.124^***^	0.004
*F*	48.29^***^	61.19^***^	41.59^***^

^***^p < 0.001; interaction term *p* = 0.151 (not significant).

All continuous variables were mean-centered prior to computation of interaction terms.

95% CIs are bias-corrected bootstrap estimates based on 5,000 resamples.

## 5 Discussion

### 5.1 The positive association between perceived barriers and cognitive antecedents

As hypothesized, perceived barriers exhibited significant positive associations with both attitude and PBC. This finding is consistent with the broader logic of constraint negotiation theory (Jackson et al., 1993), which proposes that leisure constraints do not necessarily suppress participation-related cognitions. Prior empirical evidence has demonstrated that among individuals with strong participation motivation, perceived barriers and negotiation effort are positively correlated, such that heightened barrier awareness is accompanied by greater negotiation willingness (Kim et al., 2022). In the present context, respondents who perceived greater obstacles to winter sport participation simultaneously reported more favorable attitudes and stronger behavioral control perceptions. One possible interpretation, consistent with constraint negotiation theory, is that these respondents may have been engaged in active appraisal and coping processes in which barriers functioned as mobilizing stimuli rather than deterrents. This interpretation is consistent with evidence from Chinese ice and snow sport participants suggesting that perceived barriers can trigger proactive strategies that ultimately facilitate participation intention (Liang et al., 2025). The observed positive associations are compatible with, but do not directly verify, a constraint-negotiation account because negotiation strategies were not measured.

However, it is critical to acknowledge that the present study did not directly measure negotiation strategies, coping mechanisms, adaptation processes, or resource mobilization behaviors. The data therefore demonstrate associations between perceived barriers and cognitive antecedents but do not provide empirical evidence regarding how or whether participants negotiated those barriers. The term “constraint negotiation” in the context of this study refers to a theoretical framework that may help interpret the observed pattern of associations, rather than to an empirically verified psychological process occurring within participants. Alternative explanations for the positive barrier-cognition associations warrant consideration. First, the convenience sampling approach likely overrepresented individuals with pre-existing interest in or awareness of winter sports; respondents with greater familiarity with winter sport contexts may possess sharper awareness of the associated barriers while simultaneously maintaining stronger participation motivation, producing a selection-based positive association. Second, respondents already interested in winter sports may perceive barriers differently from non-participants, reflecting differential exposure rather than differential coping. Third, social influence processes—in which exposure to peers who participate in winter sports simultaneously heightens both barrier awareness and favorable cognitions—could account for the observed positive associations. Fourth, previous sport involvement or general physical activity engagement may confer motivational predispositions that manifest as both heightened barrier salience and stronger participation-related cognitions. These alternative accounts are not mutually exclusive and may operate in conjunction with negotiation processes. Future research incorporating direct measures of negotiation strategies would be necessary to empirically adjudicate among these explanations.

### 5.2 The non-significant effect of attitude on participation intention

The finding that attitude did not significantly predict participation intention warrants careful consideration. First, attitude and subjective norm exhibited a substantial bivariate correlation (*r* = 0.661), raising the possibility that in the multivariate regression framework, subjective norm absorbed a portion of the explanatory variance that attitude might otherwise have accounted for. This interpretation aligns with discussions in the meta-analytic literature regarding collinearity risks among TPB predictors (Hagger et al., 2002). Second, the predictive validity of attitudes may be attenuated when those attitudes are formed in the absence of direct behavioral experience. Attitudes grounded in direct experience exhibit significantly stronger associations with subsequent behavior than do attitudes based on indirect or secondhand information (Fazio and Zanna, 1978; Duerden and Witt, 2010). Given that the majority of residents of the study region lack firsthand experience with winter sports, their attitudes toward these activities are likely shaped more by media representations and social discourse than by personal engagement, potentially weakening the attitude-intention link. This non-significant attitude–intention link stands in contrast to findings from traditional alpine winter sport regions, where attitude was the strongest predictor of intention (β = 0.66, *p* < 0.001) among populations with direct experiential engagement (Frühauf et al., 2020).

It is important to note that the non-significance of attitude should not be interpreted as indicating that attitude is unimportant in this context. Rather, its influence may be substantially subsumed by subjective norm. In cultural settings characterized by interdependent self-construal, the evaluations held by socially proximate others are deeply intertwined with one's own evaluative orientations, such that the conceptual boundary between social expectations and personal evaluations may be considerably more permeable than in cultural settings characterized by independent self-construal. This pattern is consistent with Chen et al. (2025), who found that core motivational variables do not directly predict leisure outcomes but operate through mediating mechanisms such as leisure involvement.

### 5.3 The primacy of subjective norm and perceived behavioral control

Subjective norm and PBC both emerged as significant predictors of participation intention among the three canonical TPB variables, with PBC exhibiting the numerically larger standardized coefficient. This pattern stands in notable contrast to the prevailing pattern in predominantly Western samples, where attitude typically dominates (mean β ≈ 0.42 to 0.51) and subjective norm tends to exhibit the weakest effect (Hagger et al., 2002). Within Chinese cultural contexts, subjective norm has been documented as a robust and consistent predictor of exercise intention (Feng et al., 2022; Li, 2023), and its predictive strength has been shown to vary as a function of cultural value orientation (Feng et al., 2023). The prominence of both subjective norm and PBC observed in the present study is consistent with the proposition that social referent group influence and behavioral control assessments carry particular weight in behavioral decisions within collectivist cultural settings. Similar patterns have been observed in East Asian winter sport contexts, where subjective norms demonstrated strong explanatory power for adolescents' participation intention (Fan et al., 2025).

It is important to emphasize that collectivism provides one plausible interpretive framework, but it was not measured in the present study and should not be treated as an empirically established explanation. The tendency to interpret findings through a collectivist cultural lens draws on well-established cross-cultural research on dominant cultural value orientations within East Asian societies, yet this aggregate cultural characterization cannot be generalized uniformly to every individual in the sample. Accordingly, arguments concerning how cultural values shape the relative predictive weights of TPB antecedents remain theoretical interpretations, which require direct empirical measurement of individual cultural values in future investigations. Readers should further note that substantial individual-level heterogeneity in value orientations exists within any broader cultural context, and aggregate-level cultural descriptors do not neatly map onto individual psychological dispositions.

### 5.4 Self-efficacy showed the numerically largest coefficient

Self-efficacy surpassed all three canonical TPB variables to emerge as the most potent predictor of winter sport participation intention (β = 0.32). This finding is consistent with Tanveer et al. (2021), who identified that self-efficacy exerted a significant positive predictive effect on behavioral intention in an extended TPB model. The present study extends this cross-domain evidence by confirming that self-efficacy retains its predictive contribution even in non-traditional winter sport contexts with high structural barriers, and further quantifies its incremental explanatory power (Δ*R*^2^ = 0.076) beyond the core TPB framework. This finding underscores the potential centrality of internal capability beliefs in decision-making contexts characterized by high uncertainty and novelty. Theoretical accounts suggest that self-efficacy assumes increasing importance for behavioral intention as situational uncertainty increases (Bandura, 1986). Residents of non-traditional winter sport regions, who generally lack familiarity with the skill demands, safety considerations, and learning trajectories associated with winter sports, may rely substantially on the self-referent question of whether they are capable of performing the activity as a primary psychological gateway to intention formation.

This finding also provides empirical support for the conceptual distinction between PBC and self-efficacy (Ajzen, 2002). In the present model, both constructs entered as independent predictors and both reached statistical significance, yet self-efficacy exhibited a larger standardized coefficient. This differential suggests that PBC and self-efficacy capture distinct facets of the perceived control construct: PBC primarily reflects appraisals of whether external conditions permit the behavior, whereas self-efficacy captures beliefs about whether the individual possesses the internal competence to perform it. In the context of a high-skill-threshold activity such as winter sport participation, internal capability beliefs may carry greater predictive weight than external condition judgments. This interpretation is consistent with findings that self-efficacy and PBC are empirically separable factors, with self-efficacy more predictive of behavioral intentions in health contexts (Parkinson et al., 2017; Povey et al., 2000).

### 5.5 Mediation pathways

The mediation analysis revealed a theoretically informative pattern: the indirect association of perceived barriers with participation intention through PBC was statistically significant, whereas the corresponding indirect association through attitude did not reach significance. This pattern suggests that perceived barriers are associated with participation intention primarily through their relationship with individuals' sense of behavioral mastery and resource sufficiency rather than through their relationship with affective or evaluative orientation. Corroborating this logic, prior research has demonstrated that structural constraints exhibit no direct impact on behavioral intentions but show significant indirect effects through cognitive mediators (Yamashita and Hallmann, 2020), although the present results suggest that this mediation operates through the behavioral control pathway rather than through affective or attitudinal channels. This finding is consistent with theoretical accounts positing that PBC assumes a particularly prominent mediating role when the focal behavior involves high execution difficulty (Ajzen, 2002). Corroborating evidence from the leisure constraint literature has similarly demonstrated that the impact of structural constraints on participation intention is primarily mediated through individuals' cognitive appraisals of constraint controllability rather than through attitudinal modification. Cross-context research further supports the primacy of control-related cognition, as internal and interpersonal constraints rather than structural barriers dominate leisure decision-making in niche and novel activity contexts (Pizzo et al., 2023).

It warrants emphasis that the cross-sectional design of this study precludes causal interpretation of these mediation pathways. The bootstrap mediation analysis tests whether the data are consistent with the hypothesized indirect effect structure, but it does not establish that perceived barriers temporally precede changes in PBC, or that changes in PBC temporally precede changes in participation intention. Future longitudinal investigations with repeated measures would be necessary to assess the causal ordering implied by the mediation model. The practical implication of this finding is that efforts to promote winter sport participation in the study region may benefit from prioritizing the enhancement of residents' sense of behavioral mastery and resource accessibility.

### 5.6 The non-significant moderating effect of self-efficacy

The hypothesized moderating role of self-efficacy in the perceived barrier-to-PBC pathway was not empirically supported. Two interpretive accounts merit consideration. First, the study sample, which was recruited through non-probability methods from individuals with pre-existing interest in winter sports, may have been characterized by relatively homogeneous levels of self-efficacy. Restricted variance in the moderator variable attenuates the statistical power to detect interaction effects. A sensitivity analysis indicated that the sample provided 80% power to detect an incremental effect of approximately *f* ^2^ = 0.019 or larger. Accordingly, smaller interaction effects may have remained undetected. Second, from a theoretical perspective, self-efficacy may not operate as a buffering or amplifying mechanism in this context but rather as an independent antecedent that directly contributes to behavioral control perceptions. The main effect of self-efficacy on PBC was substantial (β = −0.064, *p* = 0.151), suggesting that increases in self-efficacy were associated with strengthened behavioral control beliefs irrespective of the level of perceived barriers. This pattern is consistent with a direct enablement mechanism rather than a conditional moderating one.

This finding carries implications for the ongoing conceptual debate regarding the relationship between PBC and self-efficacy. In the present high-skill-threshold sport context, the two constructs functioned as empirically independent predictors, each capturing a distinct dimension of the perceived control experience: PBC addressed the question of whether conditions allow it, and self-efficacy addressed the question of whether the individual is capable of it, with the latter demonstrating greater explanatory power. Future research might productively examine these two constructs in parallel prediction models across varying task characteristics to identify the boundary conditions under which each emerges as the dominant predictor.

### 5.7 Limitations and future directions

Several limitations of the present study should be explicitly acknowledged, as they constrain the inferences that can be drawn.

First, the cross-sectional design precludes causal inference regarding the temporal ordering of variables and cannot capture the dynamic process by which intentions translate into actual behavior. The intention-behavior gap is a well-documented phenomenon in sport participation research: intentions explain approximately 28% of the variance in future behavior on average, with the gap being particularly pronounced among those holding positive intentions who nevertheless fail to act (Sheeran, 2002). The language of mediation used throughout this manuscript reflects statistical mediation in a cross-sectional design, not causal mediation requiring temporal precedence. Future investigations employing longitudinal designs with measurement occasions before, during, and after the ski season would enable more rigorous examination of the temporal predictive validity of the identified antecedents.

Second, the reliance on convenience sampling and snowball recruitment limits the external validity of the findings. The sample comprised self-identified residents of southern China, while IP-derived access locations were concentrated in Zhejiang Province. Because IP location does not necessarily correspond to permanent residence, the geographic composition of the sample should be interpreted cautiously. The findings may not generalize to southern provinces with differing levels of economic development, winter sport infrastructure, climatic conditions, or cultural orientations. Future studies employing probability-based, multi-stage stratified sampling designs across diverse southern provinces and demographic strata would enhance sample representativeness and generalizability.

Third, an a priori sample size justification was not conducted. While the final sample size (N = 415) meets general guidelines for SEM, formal power analysis would have strengthened methodological transparency. The null moderation finding for H3 may partially reflect insufficient statistical power to detect a small interaction effect. Future research should conduct a priori power analyses informed by expected effect sizes from pilot data or meta-analytic estimates.

Fourth, the present study did not incorporate measures of several potentially relevant constructs. Prior winter sport experience, frequency of physical activity participation, companion participation patterns, and specific negotiation strategies were not assessed. This restricts the comprehensiveness with which the study can characterize the complex decision-making ecology of winter sport participation. Most critically, the absence of direct measures of constraint negotiation—including coping strategies, resource mobilization behaviors, adaptation processes, or social support seeking—means that the theoretical interpretation invoking negotiation mechanisms cannot be directly tested with the current data.

Fifth, Harman's analysis and the poor fit of the single-factor CFA suggested that a single common factor did not adequately account for the observed covariance. Nevertheless, these procedures have limited sensitivity, and the absence of a theoretically appropriate marker variable prevented a stronger assessment of common method variance.

Sixth, the measurement model demonstrated acceptable fit overall, but the PBC1 item exhibited a relatively low standardized loading (0.592). Furthermore, the discriminant validity between self-efficacy and PBC, while supported by the Fornell-Larcker criterion, would benefit from additional validation through HTMT analysis and alternative CFA model comparisons.

Seventh, collectivism and other cultural value orientations were not directly measured. Interpretations invoking collectivist cultural dynamics as an explanatory framework for the observed patterns of TPB predictor weighting should therefore be regarded as plausible theoretical accounts that require direct empirical verification rather than as empirically established conclusions.

Future research should address these limitations by employing longitudinal designs, probability sampling methods, a priori power analyses, direct measures of negotiation strategies and cultural value orientations, and more robust common method bias assessments. Research that follows participants across an entire winter sport season, tracking both psychological antecedents and actual participation behavior, would be particularly valuable for bridging the intention-behavior gap identified in the current literature.

### 5.8 Future research directions

Beyond addressing the limitations noted above, several productive directions for future research emerge from this study. First, comparative studies examining the same theoretical model across multiple non-traditional winter sport regions—including other Asian countries, the Middle East, and North Africa—would shed light on the generalizability vs. cultural specificity of the observed patterns. Second, qualitative or mixed-methods investigations could illuminate the specific strategies through which residents of non-traditional winter sport regions negotiate structural, interpersonal, and intrapersonal constraints. Third, intervention studies testing whether self-efficacy enhancement programs or social norm-based promotional strategies produce measurable changes in participation behavior would bridge the gap between correlational findings and practical application. Fourth, incorporating objective measures of winter sport facility accessibility (e.g., Geographic Information System-based distance calculations) alongside self-reported barriers would strengthen the ecological validity of future investigations.

## 6 Conclusion

This study extended the TPB framework with leisure constraint theory and self-efficacy theory to examine winter sport participation intentions among residents of the Yangtze River Delta region of China. The results revealed that perceived barriers were positively associated with attitude and PBC, a pattern consistent with constraint negotiation theory, though the absence of direct negotiation measures means that the specific mechanisms underlying these associations remain to be empirically established. Among the canonical TPB predictors, PBC and subjective norm both significantly predicted participation intention, while attitude did not. Self-efficacy showed the numerically largest coefficient, although it was not statistically stronger than PBC or subjective norm, underscoring the centrality of internal capability beliefs for novel, high-threshold sport activities. The mediation analysis indicated that the indirect association of perceived barriers with intention operated through the PBC pathway rather than the attitudinal pathway, though causal interpretation is constrained by the cross-sectional design.

These findings should be interpreted in light of several key limitations: the cross-sectional design, convenience sampling with geographic concentration in Zhejiang Province, absence of a priori power analysis, and lack of direct measures of negotiation strategies and cultural value orientations. The findings provide preliminary evidence that the canonical TPB predictor hierarchy may vary across cultural contexts, that self-efficacy and PBC function as empirically distinguishable predictors in high-skill-threshold sport contexts, and that perceived barriers relate to participation intentions through behavioral control pathways. Practically, the findings suggest that promotional strategies seeking to enhance winter sport participation in non-traditional regions could benefit from prioritizing social influence mechanisms and self-efficacy enhancement.

Future research employing longitudinal designs, probability sampling, direct measurement of negotiation processes and cultural values, and robust common method bias controls is needed to substantiate and extend these findings. As winter sport participation continues to expand into non-traditional regions globally, understanding the psychological mechanisms that facilitate or inhibit engagement among populations without natural exposure to ice and snow environments remains a theoretically significant and practically consequential research priority.

## Data Availability

The original contributions presented in the study are included in the article/supplementary material, further inquiries can be directed to the corresponding author.
